# Paraoxonase 1 and Chronic Obstructive Pulmonary Disease: A Meta-Analysis

**DOI:** 10.3390/antiox10121891

**Published:** 2021-11-26

**Authors:** Jun Watanabe, Kazuhiko Kotani, Alejandro Gugliucci

**Affiliations:** 1 Division of Community and Family Medicine, Jichi Medical University, Shimotsuke 329-0498, Japan; m06105jw@jichi.ac.jp; 2 Glycation, Oxidation and Disease Laboratory, Touro University-California, Vallejo, CA 94592, USA; agugliuc@touro.edu

**Keywords:** antioxidant, arylesterase, chronic obstructive lung disease, reactive oxygen species, paraoxonase

## Abstract

Oxidative stress is a driving factor in the pathophysiology of chronic obstructive pulmonary disease (COPD). While paraoxonase 1 (PON1) is an antioxidant enzyme and a potential biomarker of this disease, data regarding the status of PON-1 in COPD are inconclusive. In this regard, to shed light on this issue, we performed a meta-analysis of data on PON1 activity in COPD. Electronic databases (MEDLINE, Embase and CENTRAL) were searched for available studies on PON1 activity in patients with stable COPD published before October 2021. A meta-analysis was performed using random-effects models. Twelve studies (12 studies on paraoxonase and three on arylesterase) were identified. Patients with COPD had lower levels of paraoxonase activity (standard mean difference [SMD] −0.77, 95% confidence interval [CI] −1.35 to −0.18) and arylesterase activity (SMD −1.15, 95% CI −1.95 to −0.36) in comparison to healthy controls. In subgroup analyses, paraoxonase activity was lower in patients of studies as consisted of mainly non-severe COPD (SMD −1.42, 95% CI −2.04 to −0.79) and, by contrast, slightly higher in patients of studies including severe COPD (SMD 0.33, 95% CI 0.02 to 0.64) in comparison to healthy controls. Arylesterase activity showed a similar trend. Overall, PON1 activity was lower in patients with COPD, suggesting that PON1-related antioxidant defense is impaired in COPD. Future studies are warranted.

## 1. Introduction

Chronic obstructive pulmonary disease (COPD), a progressive airway disorder, is a major cause of disability and death worldwide, and the number of patients is increasing [1]. Smoking and air pollution leading to increased inflammation and free radicals in the respiratory tract cause an increased burden of oxidative stress, which leads to the development and progression of COPD [2,3]. Oxidative stress is reported to induce structural changes in the essential components of the lung, including irreversible damage to both the parenchyma and airway wall [4]. In this process, various molecules, such as nucleic acids, lipids and proteins, are oxidized due to the respiratory burst of leukocytes (macrophages (Mφ) and polymorphonuclear (PMN) leukocytes [5]. Currently, oxidative stress is considered a crucial contributor to the pathophysiology of COPD [6,7,8].

The burden of oxidative stress is modified by the antioxidant balance. Paraoxonase 1 (PON1), which is known as an antioxidant enzyme, is composed of 354 amino acids with a molecular weight 43 kDa and is encoded by the *PON1* gene [9]. PON1 activity is determined by the substrates used to measure it, in particular, arylesterase (when using phenylacetate) and paraoxonase (when using paraoxon). PON1 is a high-density lipoprotein (HDL)-associated lipolactonase that has promiscuous activity as an esterase [10,11,12,13,14,15]. Based on the antioxidant properties of PON1, there have been studies regarding the roles of PON1 in various disease entities, including cardiovascular disease, kidney failure, diabetes mellitus, neurological disorders, and sleep apnea [11,12,16,17]. While the relationship between PON1 and COPD is also of interest, the current data appear inconclusive.

To date, there is no general consensus on circulating PON1 activity in patients with COPD. Given the putative importance of PON1 as a biomarker of this disease, the present study aimed to explore PON1 activity in COPD via a meta-analysis of available clinical studies.

## 2. Materials and Methods

The present review was conducted in accordance with the reporting guidelines outlined by the Preferred Reporting Items for Systematic Reviews and Meta-Analysis, PRISMA [18]. This protocol was registered in PROSPERO (ID 286949).

A search of the MEDLINE, Embase and CENTRAL electronic databases was performed. The following keywords were applied to search for studies published before 2 October 2021: ‘PON1′, ‘paraoxonase’, ‘arylesterase’ and ‘chronic obstructive pulmonary disease’ were applied to the search (Appendix A). The inclusion criteria were clinical studies that focused on PON1 activity in patients with stable COPD in comparison to healthy controls. The exclusion criteria were studies without healthy controls or studies that focused on PON1 in asthma patients. There were no restrictions on language, country, observation period, or year of publication. The reference lists of eligible studies were searched.

First, all retrieved candidate articles were independently screened according to their titles and summaries. The full texts of potentially relevant summaries were independently evaluated for eligibility. Original articles that did not focus on PON1 in patients with COPD in comparison to healthy controls were excluded. An article was considered eligible when the two researchers were in agreement. The risk of bias was evaluated using the Newcastle-Ottawa Quality Rating Scale, NOS [19]. Then, a summary table for each article was extracted and created.

Meta-analyses were performed using random-effects models in Review Manager 5.4.1 (RevMan 2020, The Nordic Cochrane Centre, Copenhagen, Denmark) [20]. The standard mean difference (SMD) and 95% confidence interval (CI) of paraoxonase and arylesterase activity were calculated. When missing data existed, standard deviations were calculated based on the methods of the Cochrane handbook [21]. Statistical heterogeneity was evaluated by visual inspection of forest plots and by calculating the *I*^2^ statistic (*I*^2^ values of 0% to 40%: May not be important; 30% to 60%: May represent moderate heterogeneity; 50% to 90%: May represent substantial heterogeneity; 75% to 100%: Considerable heterogeneity) [21]. When heterogeneity was identified (*I*^2^ statistic > 50%), the possible source of heterogeneity was examined in subgroup analyses of the severity of COPD (studies including severe COPD versus studies of mainly non-severe COPD). In the respective studies, the severity of COPD was defined according to Global Initiative for Chronic Obstructive Lung Disease criteria (https://goldcopd.org/ (accessed on 25 November 2021)) based on spirometry, symptoms, and exacerbations [21].

## 3. Results

**Figure 1:**
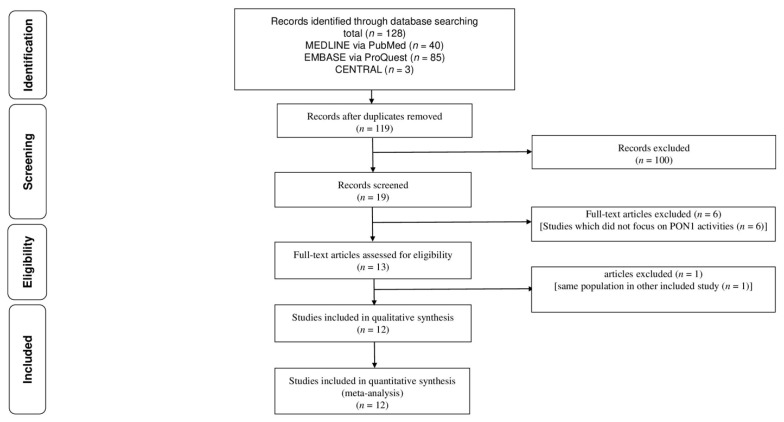
Flow of the selection of literature that reported the relationship of PON 1 with chronic obstructive pulmonary disease. PON1: paraoxonase 1.

Figure 1 shows the flow for the selection of literature that reported PON1 activity of patients with COPD. Of the 119 initially identified articles, 100 articles were excluded after screening of titles and abstracts. After screening of full-texts, six studies were excluded because six studies did not focus on PON1 activity of patients with COPD [22,23,24,25,26,27]. One study [28] was excluded because it included the same population as another study [29]. Finally, 12 studies were identified [13,14,28,29,30,31,32,33,34,35,36,37].

Table 1 shows a summary of the included studies. Of the 12 studies, 12 measured paraoxonase activity [13,14,28,29,30,31,32,33,34,35,36,37] and three measured arylesterase activity [14,32,35]. Four studies included patients with severe COPD (30–100%) [13,14,28,34]. Table 2 shows the study quality of the included studies using the NOS (median score, 7; range, 5–8).

**Figure 2:**
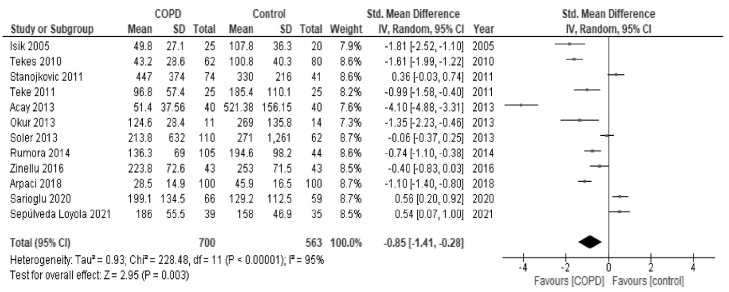
Forest plot of paraoxonase activity in all studies. COPD: chronic obstructive pulmonary disease; Std: standard; SD: standard deviation; CI: confidence interval; IV: interval variable.

**Figure 3:**
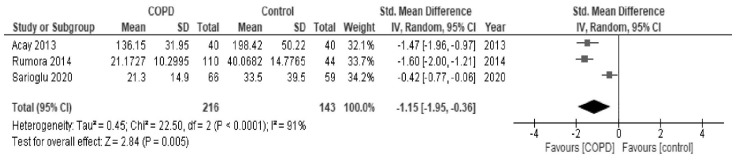
Forest plot of arylesterase activity in all studies.

In the meta-analysis, as shown in Figure 2, paraoxonase activity of patients with COPD was significantly low in comparison to healthy controls (SMD, −0.85; 95% CI, −1.41 to −0.28; *I*^2^ = 95%). As shown in Figure 3, arylesterase activity of patients with COPD was also significantly low in comparison to healthy patients (SMD, −1.15; 95% CI, −1.95 to −0.36; *I*^2^ = 91%).

**Figure 4:**
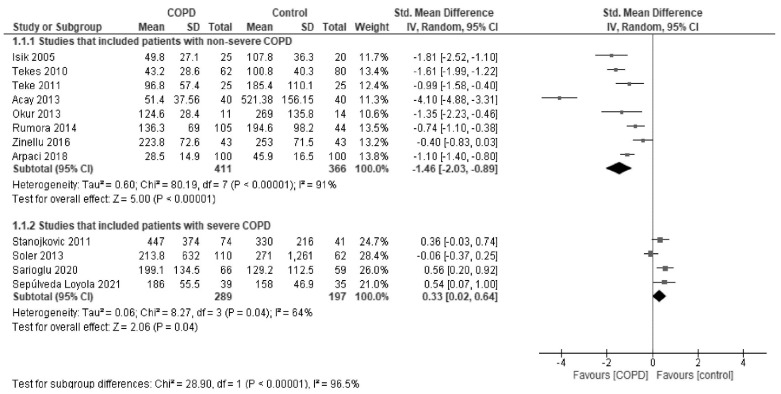
Forest plot of paraoxonase activity stratified by the severity of COPD.

**Figure 5:**
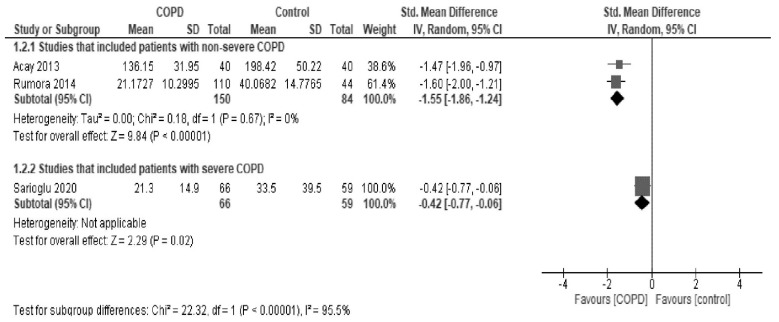
Forest plot of arylesterase activity stratified by the severity of COPD.

In subgroup analyses by the severity of COPD, paraoxonase activity was found to be significantly low in studies that mainly included patients with non-severe COPD (SMD, −1.46; 95% CI, −2.03 to −0.89; *I*^2^ = 91%) and, in contrast, was slightly but significantly higher in studies that included patients with severe COPD (SMD, 0.33; 95% CI, 0.02 to 0.64; I^2^ = 64%) in comparison to healthy controls (Figure 4). Arylesterase activity showed a similar trend (Figure 5).

**Figure 6:**
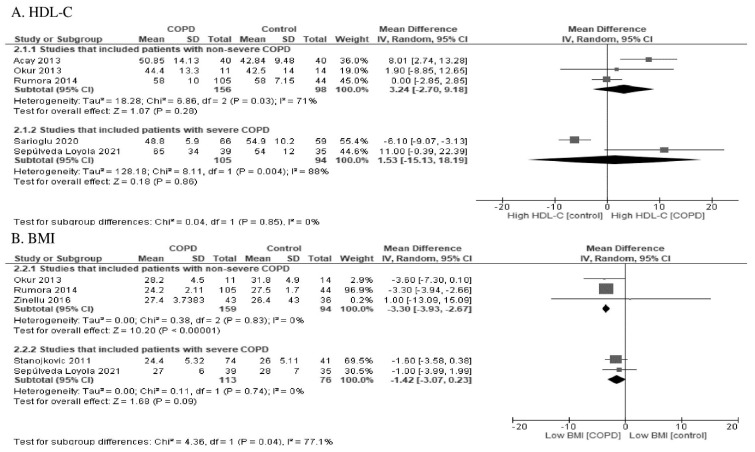
Forest plot of (**A**) high-density lipoprotein cholesterol and (**B**) body mass index stratified by the severity of COPD.

Some studies [14,28,32,33,34,35] reported the HDL-cholesterol (HDL-C) level in addition to PON1 activity. One study that included patients with severe COPD showed low levels of HDL-C [14], while another study showed high levels of HDL-C [28]. In three other studies that mainly included patients with non-severe COPD, the HDL-C levels were high [32,33,34,35]. The HDL-C levels did not differ according to the severity of COPD (test for subgroup differences: *p* = 0.85), as shown in Figure 6.

Some studies [13,28,33,35,36] reported the body mass index (BMI) in addition to PON1 activity. The BMI was higher in studies that included patients with severe COPD [13,28] in comparison to those that mainly included patients with non-severe COPD [33,35,36] (*p* = 0.04), as shown in Figure 6.

## 4. Discussion

The present study demonstrated that, overall, both paraoxonase and arylesterase activities of PON1 were lower in COPD patients in comparison to healthy controls. In addition, this lower paraoxonase activity was observed in studies that mainly included patients with non-severe COPD, but not in studies that included patients with severe COPD. Arylesterase activity also showed a similar trend. These results indicate that COPD could be generally associated with impaired PON1 activity. This implies an impaired antioxidant defense in COPD, and the measurement of PON1 activity can be useful to explore the oxidative stress-related pathophysiology of COPD.

COPD, which produces chronic oxidative stress generated by hypoxia and single electron reduction of oxygen, is hypothesized to promote both catabolism and inactivation of PON1 molecules [38,39]; accordingly, it may partly explain the low PON1 activity observed in the present study. Although paraoxonase activity (using paraoxon as a substrate) and arylesterase activity (using phenylacetate as a substrate) can differ depending on the multiple polymorphisms of PON 1 [11,12], both activities often show changes on the same direction [11,12]. Therefore, it is not surprising to find that paraoxonase and arylesterase activities show similar trends in COPD.

The present study raises the possibility that mild- and moderate-grade COPD may be associated with lower PON1 activity and paradoxically, severe COPD might be associated with slightly higher activity. Although the reason why PON1 activity differed according to the severity of COPD are unclear, we can suggest as a main explanation the status of alveolar infiltration of Mφ and PMNs which is higher in earlier stages. Indeed, myeloperoxidase (MPO) stemming from these cells is a critical PON1 inactivator [40,41]. In later stages of COPD when most parenchyma is destroyed, one expects less contact of PON1 with MPO, then less inactivation. As smoking cessation increases the levels of HDL-C and PON1 due to relief of the oxidative burden [42], patients with severe COPD could fare better due to smoking cessation Another possibility is that when the disease reaches a severe state with compromised hematosis, a compensatory effect on PON1 synthesis might ensue. Patients with severe COPD generally have oxygen therapy, an antioxidant therapy [21], which could prevent enhancement of catabolism and inactivation of PON1. Finally, as mortality increases with the progression of the severity of COPD [43], patients with severe COPD who have relatively higher PON1 activities may survive (called the ‘survival effect’ or ‘reverse causality’). According to the results of the present study and our hypotheses, further studies are needed to examine the relationship between PON1 and the severity of COPD.

The present study has some limitations. Although we searched the three main electronic databases, the number of studies included in this review was relatively small. PON1 activity is affected by lifestyle factors, such as diet and exercise. None of the studies included in the present meta-analysis examined the effects of lifestyle. Although PON1 activity is affected by polymorphism [11,12], no studies on polymorphisms were found. There are ethnic differences in lifestyle factors and polymorphisms, but we had insufficient ethnic data in the present study to confirm this issue. Stratification of COPD by severity (including studies of severe COPD versus studies of mainly non-severe COPD) may be flawed since the studies included did not always determine objectively the severity of the patients. Cohort studies and intervention studies were not performed to investigate the relationship of PON1 with COPD. The range of PON1 measurements was large because of poor standardization of methods. Since the low PON1 activity found in the present study, may be the result of low PON1 protein mass, no studies that measured PON1 mass were found. This will be addressed in future studies.

## 5. Conclusions

The present study revealed, via a meta-analysis, that, overall, PON1 activity was lower in patients with COPD. This suggests an impaired PON1-related antioxidant defense; therefore, PON1 activity can become a useful biomarker for the assessment of the oxidative stress burden in COPD. PON1 activity may be used for COPD management if data regarding a prognostic dimension of PON1 are accumulated in patients with COPD. Future studies are called for.

## Figures and Tables

**Table 1:** Summary of the included articles on PON1 activity in patients with COPD.

Authors [Ref No.]	Year	Country	Subject No.	Age	Activity in COPD	Activity in Healthy Controls	Included Severe COPD (%)
**Paraoxonase**							
Isik [29]	2005	Turkey	45	61	49.8 ± 27.1	107.8 ± 36.3	NR
Tekes [30]	2010	Turkey	62	60	43.2 ± 28.6	100.8 ± 40.3	NR
Stanojkovic [13]	2011	Serbia	74	63	447 ± 337	330 ± 216	100
Teke [31]	2011	Turkey	25	63	96.8 ± 57.4	185.4 ± 110.1	NR
Acay [32]	2013	Turkey	40	62	51.4 ± 37.5	521.4 ± 156.2	NR
Okur [33]	2013	Turkey	11	57	124.6 ± 28.4	269.0 ± 135.8	NR
Soler [34]	2013	Spain	110	NR	213.8 ± 632.0	271.0 ± 1261.0	38
Rumora [35]	2014	Croatia	105	71	136.3 ± 69.0	194.6 ± 98.2	NR
Zinellu [36]	2016	Italy	43	74	223.8 ± 72.6	253.0 ± 71.5	0
Arpaci [37]	2018	Turkey	100	NR	28.5 ± 14.9	45.9 ± 16.5	NR
Sarioglu [14]	2020	Turkey	66	64	199.1 ± 134.5	129.2 ± 112.5	30.3
Sepúlveda Loyola [28]	2021	Brazil	39	69	186.0 ± 55.5	158 ± 46.9	54
**Arylesterase**							
Acay [32]	2013	Turkey	40	62	136.2 ± 32.0	198.4 ± 50.2	0
Rumora [35]	2014	Croatia	105	71	21.2 ± 10.3	40.1 ± 14.8	NR
Sarioglu [14]	2020	Turkey	66	64	21.3 ± 14.9	33.5 ± 39.5	30.3

COPD, chronic obstructive pulmonary disease; NR, not reported; PON1, paraoxonase 1.

**Table 2:** Study quality of the included studies.

Authors [Ref No.]	The Newcastle-Ottawa Quality Assessment Scale								
	Selection				Comparability	Outcome			Total
	Representatives of the Exposed Cohort/Adequate Case Definition (0, 1)	Selection of the Non-Exposed Cohort/ Representative of Cases (0, 1)	Ascertainment of Exposure/ Selection of Controls (0, 1)	Demonstration That Outcome of Interest Was Not Present at Start of Study/Definition of Controls (0,1)	Comparability on the Basis of Design or Analysis (0, 1, 2)	Assessment of Outcome/Exposure (0, 1)	Was Follow-Up Long Enough for Outcomes to Occur (0, 1)	Adequacy of Follow-Up of Cohorts (0, 1)	Score
Isik [29]	1	0	0	0	1	1	1	1	5
Tekes [30]	1	0	0	0	1	1	1	1	5
Stanojkovic [13]	1	1	1	1	1	1	1	1	8
Teke [31]	1	0	0	0	1	1	1	1	5
Acay [32]	1	0	1	1	1	1	1	1	7
Okur [33]	1	0	1	1	1	1	1	1	7
Soler [34]	1	1	1	0	1	1	1	1	7
Rumora [35]	1	0	1	1	1	1	1	1	7
Zinellu [36]	1	1	1	1	1	1	1	1	8
Arpaci [37]	1	0	1	0	1	1	1	1	6
Sarioglu [14]	1	1	1	1	1	1	1	1	8
Sepúlveda Loyola [28]	1	1	1	1	1	1	1	1	8

## Appendix A

MEDLINE via PubMed

#1. “Aryldialkylphosphatase”[Mesh]

#2. “aryldialkylphosphatase”[tiab]

#3. “arylesterase”[tiab]

#4. “paraoxonase”[tiab]

#5. #1 OR #2 OR #3 OR #4

#6. “Lung Diseases, Obstructive”[Mesh]

#7. “Pulmonary Disease, Chronic Obstructive”[Mesh]

#8. emphysema*[tiab]

#9. chronic*[tiab] AND bronchiti*[tiab]

#10. obstruct*[tiab] AND (pulmonary[tiab] OR lung*[tiab] OR airway*[tiab] OR aiflow*[tiab] OR bronch*[tiab] OR respirat*[tiab])

#11. COPD[tiab] OR COAD[tiab] OR COBD[tiab] OR AECB[tiab]

#12. #6 OR #7 OR #8 OR #9 OR #10 OR #11

#13. #5 AND #12

CENTRAL via Cochrane Library

#1. MeSH descriptor: [Aryldialkylphosphatase] explode all trees

#2. aryldialkylphosphatase:ti,ab,kw (Word variations have been searched)

#3. arylesterase:ti,ab,kw (Word variations have been searched)

#4. paraoxonase:ti,ab,kw (Word variations have been searched)

#5. #1 OR #2 OR #3 OR #4

#6. MeSH descriptor: [Lung Diseases, Obstructive] explode all trees

#7. MeSH descriptor: [Pulmonary Disease, Chronic Obstructive] explode all trees

#8. emphysema*:ti,ab,kw (Word variations have been searched)

#9. (chronic* AND bronchiti*):ti,ab,kw (Word variations have been searched)

#10. (obstruct* AND (pulmonary OR lung* OR airway* OR airflow* OR bronch* OR respirat*)):ti,ab,kw (Word variations have been searched)

#11. (COPD OR COAD OR COBD OR AECB):ti,ab,kw (Word variations have been searched)

#12. #6 OR #7 OR #8 OR #9 OR #10 OR #11

#13. #5 AND #12

Embase via Proquest

S1 EMB.EXACT.EXPLODE(“aryldialkylphosphatase”)

S2 ab(aryldialkylphosphatase) OR ti(aryldialkylphosphatase)

S3 ab(arylesterase) OR ti(arylesterase)

S4 ab(paraoxonase) OR ti(paraoxonase)

S5 S1 OR S2 OR S3 OR S4

S6 EMB.EXACT.EXPLODE(“obstructive lung disease”)

S7 EMB.EXACT.EXPLODE(“chronic obstructive lung disease”)

S8 ab(emphysema*) OR ti(emphysema*)

S9 EMB.EXACT.EXPLODE(“chronic obstructive lung disease”)

S10 ab(emphysema*) OR ti(emphysema*)

S11 (ab(chronic*) OR ti(chronic*)) AND (ab(bronchiti*) OR ti(bronchiti*))

S12 (ab(obstruct*) OR ti(obstruct*)) AND ((ab(pulmonary) OR ti(pulmonary)) OR (ab(lung*) OR ti(lung*)) OR (ab(airway*) OR ti(airway*)) OR (ab(bronch*) OR ti(bronch*)) OR (ab(respirat*) OR ti(respirat*)))

S13 (ab(COPD) OR ti(COPD)) OR (ab(COAD) OR ti(COAD)) OR (ab(COBD) OR ti(COBD)) OR (ab(AECB) OR ti(AECB))

S14 S6 OR S7 OR S8 OR S9 OR S10 OR S11 OR S12 OR S13

S15 S5 AND S14
